# Evaluation of a New Riluzole‐Based Compound VA945 on Sodium and Potassium Conductances Expressed by SH‐SY5Y‐ Derived Neurons

**DOI:** 10.1111/jnc.70280

**Published:** 2025-10-31

**Authors:** J. Cazzola, F. Talpo, G. Faravelli, C. Donati, S. Maramai, M. Saletti, G. Giuliani, M. Paolino, A. Cappelli, M. Anzini, P. Sommi, A. Vitali, A. Sala, A. Trucco, G. R. Biella, P. Spaiardi

**Affiliations:** ^1^ Department of Biology and Biotechnology “Lazzaro Spallanzani” University of Pavia Pavia Italy; ^2^ Department of Biotechnology, Chemistry and Pharmacy University of Siena Siena Italy; ^3^ Department of Molecular Medicine, Human Physiology Unit University of Pavia Pavia Italy; ^4^ Department of Chemistry University of Pavia Pavia Italy; ^5^ SC Coordination, Support and Monitoring of Clinical and Experimental Research IRCCS Foundation Policlinico San Matteo Pavia Italy; ^6^ Department of Molecular Medicine, Biochemistry Unit University of Pavia Pavia Italy; ^7^ Istituto Nazionale di Fisica Nucleare Sezione di Pavia Pavia Italy

## Abstract

Riluzole (Rilutek), a derivative of benzothiazole, acts as a neuroprotective agent by inhibiting voltage‐dependent sodium (Na^+^) and delaying rectifier potassium (K^+^) currents. By doing so, it helps reduce excitotoxicity, a key pathogenetic mechanism in various neurodegenerative diseases, including amyotrophic lateral sclerosis (ALS). Although riluzole is a clinically approved treatment for ALS, it is not fully effective, particularly in advanced stages of the disease. In this study, we functionally characterized a newly synthetized riluzole‐based compound, VA945, with potentially enhanced neuroprotective effects. By means of SH‐SY5Y human neuroblastoma cells differentiated into neurons, we assessed using whole‐cell patch‐clamp techniques the effects of VA945 on voltage‐dependent Na^+^ and K^+^ currents at extracellular concentrations of 5, 50, and 100 μM. The compound reduced maximal activation and inactivation of Na^+^ conductance, as well as maximal activation of K^+^ conductance, across all tested concentrations. We also observed shifts of the activation and inactivation curves to more hyperpolarized potentials along with changes in the slope factor (*k*), indicating an altered voltage sensitivity of voltage‐dependent K^+^ and Na^+^ channels. While the activation kinetics of both channels remained unaffected, and the inactivation kinetics of Na^+^ were unchanged, we noted a slowdown in the deactivation kinetics of the K^+^ channels. Altogether, these findings suggest that VA945 exerts multi‐target pharmacological effects on neuronal voltage‐dependent ion currents critically involved in excitotoxicity and neurodegeneration, across a wide range of concentrations. This warrants further ex vivo and/or in vivo studies to explore its potential as a neuroprotective agent.

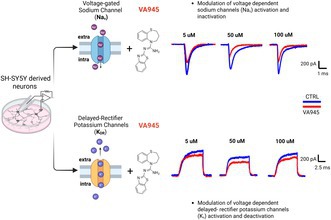

## Graphical abstract

The potential capability of a newly synthesized riluzole‐based compound, VA945, to affect neuronal excitability was tested by assessing its ability to modulate the biophysical properties of voltage‐gated sodium (Na_v_) and Delayed‐Rectifier Potassium (K_DR_) channels expressed in SH‐SY5Y human neuroblastoma cells differentiated into neurons. Using the whole‐cell patch‐clamp technique applied to these neurons, it was demonstrated that VA945, at different concentrations, exhibits multi‐target activity. Specifically, it significantly decreases the amplitude and modulates the voltage‐dependence and kinetics, including Na_v_ activation/inactivation and K_DR_ activation/deactivation, of both voltage‐gated Na^+^ and K^+^ currents. These pharmacological effects on key neuronal ionic conductances suggest that VA945 has therapeutic potential against excitotoxicity and neurodegeneration.

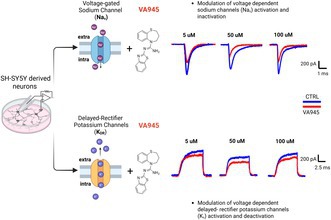

**Abbreviations**
AD: Alzheimer's disease
ALS: amyotrophic lateral sclerosis
AMPA: α‐amino‐3‐methyl‐4‐isoxazoleproprionic acid
AP: action potential
BBB: blood–brain barrier
BDNF: brain‐derived neurotrophic factor
C_m_
: membrane capacitance
CNS: central nervous system
CsCl: cesium chloride CsCl
DF: degree of freedom
DMEM: Dulbecco modified eagle medium
DMSO: dimethyl sulfoxide
E_K_
: potassium reversal potential
E_Na_
: sodium reversal potential
FBS: Fetal bovine serum
G_max_
: Maximal conductance
G_peak_
: conductance
I_A_
: transient A‐type K(+) current
I_NaL_
: late sodium current
I_NaP_
: persistent sodium current
J_Na_
: sodium current density
*k*
: slope factor
NDDs: Neurodegenerative diseases
NiCl_2_
: nickel chloride
NMDA: *N*‐methyl‐D‐aspartate
NO: nitric oxide
PD: Parkinson's disease
RA: retinoic acid
R_in_
: input resistance
ROS: reactive oxygen species
RRID: research resource identifier
R_s_
: series resistance
TEA‐Cl: tetraethylammonium chloride
TTX: tetrodotoxin
V_1/2_
: half maximal potential
V_hold_
: holding potential
V_test_
: tested membrane potential
τ_d_
: potassium deactivation time constant
τ_h_
: sodium inactivation time constant
τ_m_
: sodium activation time constant
τ_n_
: potassium activation time constant

## 1 Introduction

Neurodegenerative diseases (NDDs) involve the gradual breakdown of neuronal function due to the degeneration of synapses, axons, and nerve cells. This gradual decline disrupts neural networks and leads to impairments, in sensory, motor, and cognitive functions (Andreone et al. 2020; Wilson et al. 2023). Neuronal loss may arise from acute insults, such as traumatic brain injuries or stroke, or by chronic conditions, such as Alzheimer's disease (AD), Parkinson's disease (PD), and amyotrophic lateral sclerosis (ALS). Aging is the primary risk factor for most NDDs and exacerbates the prevalence and the impact of these pathologies, particularly within the growing elderly population, who are often burdened with multiple comorbidities. This contributes to significant human suffering and economic costs (Hou et al. 2019). Chronic NDDs are typically identified by specific pathological protein aggregations and progressive loss of selectively vulnerable populations of neurons. However, they also have a complex multifactorial etiology involving intricate interactions among genetic, molecular, and cellular mechanisms. These converging pathways ultimately lead to a common final endpoint: neurodegeneration (Dugger and Dickson 2017).

There is a range of evidence suggesting that overstimulation of excitatory receptors may cause brain damage in both acute injuries and chronic neurodegenerative conditions (Armada‐Moreira et al. 2020; Lewerenz and Maher 2015). In excitotoxicity, excessive release of glutamate from the presynaptic neurons leads to the overactivation of the postsynaptic glutamate receptors. This leads to a dysregulation of intracellular calcium (Ca^2+^) homeostasis and stimulates the production of free radicals and nitric oxide (NO). These molecules are responsible for oxidative stress, mitochondrial dysfunction, and eventually neuronal cell death. Glutamate‐induced excitotoxicity plays an important role in motor neuron degeneration observed in ALS (Arnold et al. 2024; Corona et al. 2007; Odierna et al. 2024). Cation channels are also involved in the development of excitotoxicity. In particular, the activation of voltage‐gated Na^+^ channels in presynaptic neurons is directly responsible for action potential (AP) propagation along the axon, enabling the release of glutamate implicated in excitotoxic damage (Farber et al. 2002; Piña‐Crespo, 2014). Once released, glutamate binds to the *N*‐methyl‐D‐aspartate (NMDA) and α‐amino‐3‐methyl‐4‐isoxazoleproprionic acid (AMPA) receptors on postsynaptic neurons, promoting the influx of cations and disrupting the osmotic balance. This results in excitotoxic swelling of the neurons (Choi 2020; Dong et al. 2009). This phenomenon is further exacerbated by depolarization‐induced opening of voltage‐gated Na^+^ channels on postsynaptic neurons. Voltage‐gated K^+^ channels are also involved in apoptosis, an event that may be caused by neuronal hyperexcitability (Elmogheer 2023; Pal et al. 2003; Yu 2003). Apoptotic cell death is often accompanied by a reduction in cell volume, a phenomenon known as apoptotic volume decrease, which involves different voltage‐gated K^+^ channels. Notably, the delayed‐rectifier K^+^ current is enhanced during apoptosis, even before the activation of caspases (Pasantes‐Morales and Tuz 2006). These observations underscore the potential of targeting cation channels, including voltage‐gated Na^+^ and K^+^ channels, with multimodal pharmacological strategies. This approach aims to counteract the excitotoxic processes involved in the pathogenesis of several NDDs (Binvignat and Olloquequi 2020).

**FIGURE 1:**
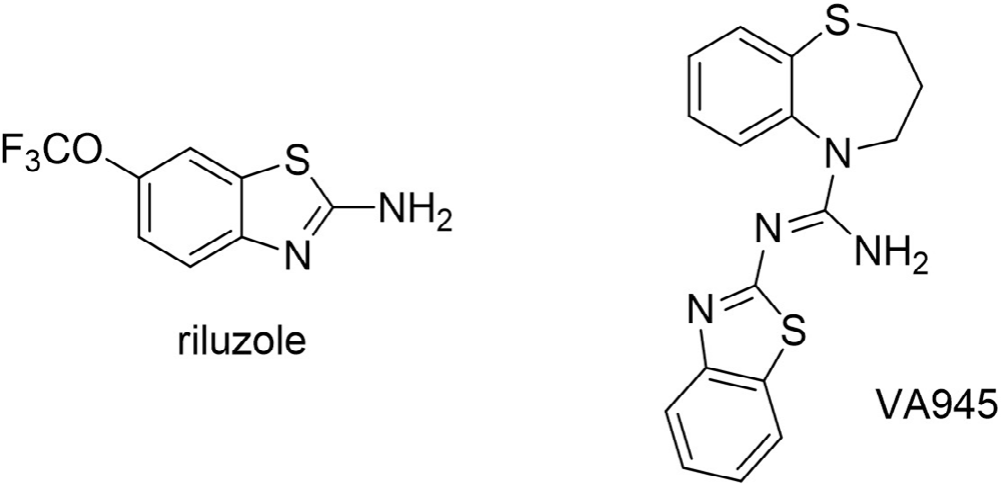
Chemical structure of riluzole and the newly developed neuroprotective agent VA945.

Riluzole (Rilutek, Figure 1) and edaravone are currently the only clinically approved treatments for ALS, a neurodegenerative disease in which excitotoxicity plays a critical role in its pathogenesis (Albertini et al. 2022; Armada‐Moreira et al. 2020). While edaravone has been demonstrated to act as an antioxidant on mitochondrial dysfunction (Cha and Kim 2022), the effect of riluzole is mainly due to its modulatory action on voltage‐gated Na^+^ and K^+^ channels. Riluzole rapidly inactivates voltage‐dependent Na^+^ currents, thereby inhibiting the release of glutamate and serving as both a neuroprotective and anticonvulsant drug (Bellingham 2011). The pharmacological effects of riluzole on ion currents are concentration‐dependent. At low concentrations (< 10 μM), it decreases the amplitude of tetrodotoxin (TTX)‐sensitive Na^+^ current without affecting the voltage‐dependence of activation and inactivation (Bellingham 2011; Beltran‐Parrazal and Charles 2003). Higher concentrations (between 0.1 and 1 mM) are required to alter the voltage‐dependence of inactivation (Benoit and Escande 1991; Hebert et al. 1994). Additionally, at concentrations above 50 μM, riluzole inhibits delayed‐rectifier K^+^ currents leading to a decreased firing frequency and further reduction of glutamate release (Bellingham 2011; Sankaranarayanan et al. 2009; Zona et al. 1998).

Due to the non‐linear dose–response relationship and limited efficacy of riluzole at certain concentration ranges, researchers' efforts have been focused on identifying new compounds with improved pharmacological properties. To this aim, the benzothiazole scaffold of riluzole has been exploited to develop novel agents with similar multi‐target mechanisms of action. In recent years, Anzini and co‐workers have proposed a variety of riluzole derivatives, including amidine, guanidine, and thiourea analogs, as neuroprotective agents for the treatment of brain diseases (Anzini et al. 2010). Similarly, the benzothiazine core has been used by the same group to generate new analogs with enhanced neuroprotective effects (Mancini et al. 2017). Recently, a small class of agents based on the benzo[*b*][1,4]thiazepine nucleus was serendipitously obtained during attempts to synthesize homodimers of riluzole (Maramai et al. 2024). Preliminary in vitro studies pointed out their potential as neuroprotective agents with multi‐target activity, thereby raising interest around this class of compounds. In this study, we tested the most promising agent, namely VA945 (Figure 1), focusing on its effects on the same ionic currents targeted by riluzole. Our aim was to elucidate its mechanism of action and evaluate its therapeutic potential.

## 2 Material and Methods

### 2.1 Synthesis of Riluzole and Title Compound VA945

VA945 and riluzole were synthesized according to previously reported procedures (Maramai et al. 2024). Their purity was assessed by LC–MS analysis and found to be higher than 95%.

### 2.2 Cell Differentiation

All experiments were conducted on SH‐SY5Y‐derived neurons. The SH‐SY5Y cell line (RRID:CVCL_0019) is not listed as a commonly misidentified cell line according to the International Cell Line Authentication Committee (ICLAC). Cell line authentication has not been performed. For this study the maximum number of passages for the cell line is 26. Cells were maintained at 37°C in a humidified atmosphere with 5% CO_2_. Undifferentiated cells have grown in Dulbecco Modified Eagle Medium (DMEM:F12, Euroclone, cat. no. ECM0096L) supplemented with 10% heat‐inactivated fetal bovine serum (FBS, South America origin EU Approved 500 mL, Euroclone, cat. no. ECS0180L), 1× Pen/Strep (Euroclone, cat. no. ECB3001D), and 2 mM Glutamine (Euroclone, cat. no. ECB3000D). After cell seeding, they were differentiated with one of the two following protocols.

**FIGURE 2:**
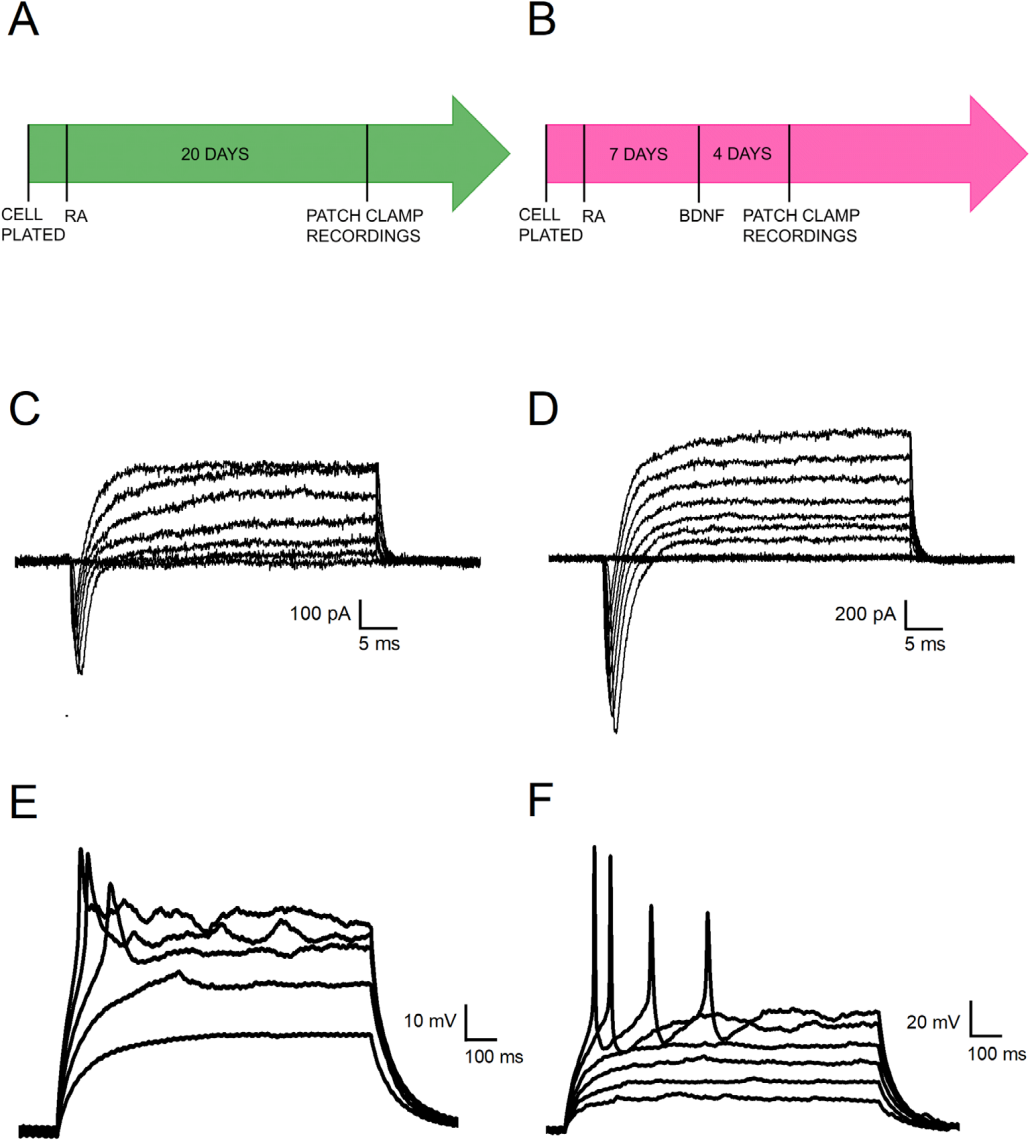
Differentiation of SH‐SY5Y cells toward a neuronal phenotype with two different protocols. (A, B) Schematic representation of the two differentiation protocols. In the first protocol (A) cells were treated for 20 days with RA (RA), while in the second one (B) cells were treated for 7 days with RA followed by 4 days with BDNF (RA + BDNF). (C, D) Representative inward and outward currents recorded in voltage‐clamp mode from cells differentiated with RA (C) and with RA + BDNF (D). Cells were tested at potential ranging between −70 and +50 mV from a holding potential of −90 mV. (E, F) Representative voltage responses from cells subjected to two different differentiation protocols, following the injection of sub‐threshold and supra‐threshold depolarizing current steps. Cells treated with RA exhibited abortive APs (E), while cells treated with RA + BDNF were able to generate single APs or sustained firing (F).

- The first differentiation protocol (Figure 2A) required the treatment with 10 μM all trans Retinoic Acid (RA, Sigma, cat. no. R2625) for 20 days. This treatment was added to a culture medium composed of DMEM:F12, 0.5% heat‐inactivated FBS, 1× Pen/Strep, and 1× glutamine (Toselli et al. 1991).
- The second differentiation protocol (Figure 2B) lasted 12 days, during which cells were treated with 10 μM RA for 7 days and then 50 ng/mL Human BDNF (Peprotech, cat. no. 450‐02‐1 mg) for 4 days. In particular, on the day after the seeding, undifferentiated cells started the differentiation with 10 μM RA added to DMEM:F12, 0.5% heat‐inactivated FBS, 1× Pen/Strep, and 2 mM Glutamine. On day eight, with the medium was changed to DMEM: F12, 1× Pen/Strep, and 2 mM Glutamine supplemented with 50 ng/mL brain‐derived neurotrophic factor (BDNF) (de Meiros et al. 2019).

### 2.3 Patch Clamp Experiments

Whole‐cell patch‐clamp experiments were performed on SH‐SY5Y‐derived neurons at room temperature (23°C–25°C). Cells were visualized using an inverted microscope (Nikon Eclipse TE200) equipped with 10X and 40X objectives, as previously described (Binini et al. 2017; Conforti et al. 2022). Recordings were conducted in both voltage‐clamp and current‐clamp modes with an Axopatch 200B amplifier (RRID:SCR_018866, Axon instruments Inc., Burlingame, CA, USA) and DigiData 1322A (RRID:SCR_021041, Molecular Devices, Sunnyvale, CA, USA). Different pipette and bath saline solutions were used depending on the type of experiment (Table 1). Patch pipettes were produced from borosilicate glass capillary tubes (Hilgenberg GmbH, Malsfeld, Germany) by using a horizontal puller (P‐97, Sutter instruments) and their resistance was 4–5 MΩ when filled with the intracellular solution. The pipette resistance was kept as low as possible, despite the greater difficulty in obtaining a gigaseal, to minimize the series resistance (*R*_s_). Membrane potentials were not corrected for residual series resistance (R_s_, 12.96 ± 1.29 MΩ, *n* = 72). *R*_s_ and the cell membrane capacitance were calculated off‐line by the capacitive artifact elicited by a voltage step from −70 to −80 mV. The calculated liquid junction potential with these solutions was about 15.5 mV (combination 1), 3.5 mV (combination 2) and 3.5 mV (combination 3). The data presented were not corrected for the calculated liquid junction potential. Series resistance (R_s_) was not compensated. Linear leak subtraction, based on the resistance estimated by 4 hyperpolarizing pulses (P/4) applied before the depolarizing test potential, was used for all voltage‐clamp recordings. Data were acquired using software Clampex (Molecular Devices, Sunnyvale, CA, USA, RRID:SCR_011323), sampled at 50 kHz, and low‐pass filtered at 10 kHz. Compound VA945 was dissolved in dimethyl sulfoxide (DMSO, Sigma, cat. no. D5879) at a concentration of 30 mM and stored at −20°C as previously described (Anzini et al. 2010). To test its effect on the electrophysiological properties of the cells, the aliquots were diluted to the final concentrations (5, 50, 100 μM) in the proper extracellular solution and focally perfused using a multi‐barrel pipette, which allowed the rapid effects of the drug to be observed within a time frame of < 2 s. The highest final concentration of DMSO used in the experiments was 0.3% v/v, introduced as a vehicle for VA945 at a final concentration of 100 μM.

**TABLE 1:** Composition of pipette and bath solutions used for patch clamp recordings.

		Pipette solution (in mM)	Bath solution (in mM)
Type of the experiment	(1) I_tot_	K‐gluconate (130, Sigma, cat. no. G4500), NaCl (4, Sigma, cat. no. S9888), MgCl_2_ (2, Sigma, cat. no. M2670), EGTA (1, Sigma, cat. no. E4378), HEPES (10, Sigma, cat. no. H3375), phosphocreatine (5, Sigma, cat. no. P7936), Na_2_ATP (2, Sigma, cat. no. A6419), Na_3_GTP (0.3, Sigma, cat. no. G8877) (pH 7.3 with KOH, Sigma, cat. no. P1767)	NaCl (140), MgCl_2_ (1), CaCl_2_ (2, Sigma, cat. no. C3881), KCl (3, Sigma, cat. no. P3911), glucose (10, VWR, cat. no. 101174Y), HEPES (10) (pH 7.4 with NaOH, Sigma, cat. no. S8045)
	(2) I_Na_	CsCl (120, Sigma, cat. no. C4036), NaCl (10), TEA‐Cl (20, Sigma, cat. no. T2265), EGTA (10), HEPES (10), MgCl_2_ (2) (pH 7.3 with CsOH, Sigma, cat. no. 232068)	NaCl (140), KCl (3), MgCl_2_ (1), CaCl_2_ (1), HEPES (10), NiCl_2_ (0.6, Sigma, cat. no. 339350), TEA‐Cl (10) (pH 7.4 with NaOH)
	(3) I_K_	KCl (128), NaCl (10), MgCl_2_ (2), CaCl_2_ (1), EGTA (11), HEPES (10) (pH 7.25 with KOH)	NaCl (155), KCl (3), MgCl_2_ (1), CaCl_2_ (1), NiCl_2_ (0.6), HEPES (10), TTX (0.001, Bio‐techne, cat. no. 1078/1) (pH 7.35 with NaOH)

*Note:* The combination of solutions 1 mimics the physiological composition of intra‐/extra‐cellular environment. The combination of solutions 2 allows isolation of sodium current: Cesium chloride (CsCl) and Tetraethylammonium chloride (TEA‐Cl) block K^+^ channels and Nickel chloride (NiCl_2_) blocks voltage‐gated Ca^2+^ channels. The combination of solutions 3 allows isolation of potassium current: Tetrodotoxin (TTX) blocks voltage‐gated Na^+^ channels and Nickel chloride (NiCl_2_) blocks voltage‐gated Ca^2+^ channels. All chemicals were from Sigma‐Aldrich, except for TTX which was from Alomone Labs.

### 2.4 Data Analysis

Data analysis was carried out with Clampfit 10.7 (Molecular Devices, Sunnyvale, CA, USA) and Origin 2018 (Microcal Software Inc., Northampton, MA, USA).

In voltage‐clamp mode, cell membrane capacitance (C_m_) was calculated by integrating the peak of the capacitive current evoked by a voltage step from −70 to −80 mV. This value was then divided by the delta of the voltage step (−10 mV). Input resistance (R_in_) was calculated from the same protocol, as the ratio between the step of voltage (−10 mV) and the value of the current trace at the end of the 35 ms pulse. Inward and outward currents were evoked by 10 mV depolarizing steps (40 ms each) from a V_hold_ of −90 mV, ranging from −70 to +50 mV. The inward current was measured for each cell at the maximum peak of negative current, while the outward current was measured at steady state, in response to a + 50 mV step. In current‐clamp mode, the presence or absence of APs was determined by the injection of supra‐threshold current steps from a holding potential (V_hold_) of −70 mV. All these parameters were derived in the presence of combination 1 of pipette/bath solutions (Table 1).

The analysis of the isolated voltage‐gated currents expressed by SH‐SY5Y‐ derived neurons was performed thanks to dedicated protocols in voltage‐clamp mode.

Voltage‐gated Na^+^ currents were isolated by using combination 2 of pipette/bath solutions (Table 1) and applying a protocol composed of +5 mV depolarizing steps (40 ms each), ranging from −70 to +50 mV, starting from a V_hold_ of −90 mV (Figure 4A). The activation curve was calculated by plotting the normalized conductance (normalization for the maximal conductance value for each cell) at each test potential. Specifically, conductance (g_peak_) was defined by the formula:

(1) $$g_{\text{peak}}=J_{\mathrm{Na}}/\left(V_{\text{test}}-E_{\mathrm{Na}}\right)$$

where J_Na_ is the measured current density (i.e., the peak current normalized to C_m_), V_test_ is the tested membrane potential, and E_Na_ is the sodium reversal potential, corresponding to +66.48 mV in our ionic conditions. The inactivation curve was calculated in the same way and by using the same Equation (1), but with J_Na_ corresponding to the peak currents divided by C_m_ measured in response to a pulse to −10 mV (24 ms), preceded by conditioning pulses (40 ms) progressively increasing from −90 to 5 mV (ΔV = 5 mV) (Figure 5C). The normalized activation and inactivation curves were fitted using a Boltzmann equation in the form:
(2)

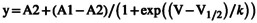

where A2 is the minimal value of normalized conductance (≈0), A1‐A2 is the normalized conductance span, V_1/2_ is the half maximal potential, and *k* is the slope factor. The activation and the inactivation curves normalized at the same maximal value were also overlapped to obtain the window currents accounting for the sodium channels availability at different membrane potentials, determined by the area subtended by the intersection of the curves (Figure 5G,H). Activation (τ_m_) and inactivation sodium time constant (τ_h_) were obtained by fitting the activation or the inactivation portion of the traces, at each test potential. The activation portion was fitted using an exponential function raised to the third power, while the inactivation portion by a standard monoexponential function. To analyze the voltage‐dependency of kinetic parameters we extrapolated the T1 parameter from a tau‐to‐voltage relationship, which was obtained and fitted with an exponential decay function in the form:

(3) $$y=\text{y0}+A_{1}e^{-x/\text{T1}}$$

Voltage‐gated delayed‐rectifier K^+^ currents were isolated by using the combination 3 of pipette/bath solutions (Table 1). We applied a protocol consisting of depolarization steps (16 ms) of 5 mV, ranging from −50 to 85 mV, starting from a V_hold_ of −90 mV (Figure 7A). The activation curve was calculated by using formula (1) and fitted with the Boltzmann Equation (2), as previously described for Na^+^ current. The reversal potassium potential (E_K_) was estimated to be −94.55 mV under our ionic conditions. The activation potassium time constant (τ_n_) was obtained by fitting the activation portion of the traces with an exponential function raised to the third power and the voltage‐dependency of the activation kinetics was obtained using Equation (3), as previously described for Na^+^ current. Deactivation analysis required a dedicated protocol, consisting of a pulse to 50 mV (10 ms) followed by pulses (100 ms) iteratively incremented from −70 to 20 mV (ΔV = 5 mV), from a V_hold_ of −90 mV. To evaluate the deactivation potassium kinetics (τ_d_), the deactivation portion of the traces was fitted with a non‐linear least‐square curve fit using the following Hodgkin‐Huxley type equation (Tosetti et al. 1998):

(4) $$I=A{\left[n_{\inf}-\left(n_{\inf}-n_{0}\;\right)\;\exp\left(-t/t_{n}\right)\right]}^{3}$$

To evaluate the voltage‐dependency of the deactivation kinetics the tau‐to‐voltage relationship was fitted with a growth exponential function in the form:

(5) $$y=y0+A_{1}e^{x/T1}$$

### 2.5 Statistical Analysis

Before parametric tests were applied, normality of each dataset was assessed by applying Kolmogorov–Smirnov tests. Data were considered normally distributed if the *p*‐value was greater than 0.05. No test for outliers was conducted. Data are presented as mean ± standard error of the mean (s.e.m). Statistical significance was determined using a paired *t*‐test to compare the effect of VA945 to the control condition, while a two‐sample *t*‐test was employed to evaluate the differences between the two differentiation protocols. Differences were considered statistically significant at *p < 0.05, **p < 0.01, and ***p < 0.001. Statistical analysis was performed with Origin 2018 (Microcal Software Inc., Northampton, MA, USA).

## 3 Results

### 3.1 Assessment of SH‐SY5Y Differentiation Toward a Functional Neuronal Phenotype

Experiments were performed on SH‐SY5Y cells differentiated toward a neuronal phenotype. These cultured cells represent an excellent model for the detailed study of the biophysical properties of isolated ionic currents as shown in the wide literature (Demontis et al. 2020; Gambino et al. 2022; Santillo 2022; Tosetti et al. 1998). It has been demonstrated that SH‐SY5Y cells can be effectively differentiated into a functional neuronal phenotype through specific protocols (D'Aloia et al. 2024; de Meiros et al. 2019; Kaya et al. 2024; Şahin et al. 2021). In order to determine the most effective method to obtain functional neuronal phenotype, we compared two differentiation protocols that are schematically shown in Figure 2A,B and described in the Material and Methods section. In summary, the first group of cells was treated with retinoic acid (RA) for 20 days prior to electrophysiological experiments (Figure 2A), while the second group was treated with RA for 7 days followed by 4 days of exposure to BDNF (Figure 2B).

**FIGURE 3:**
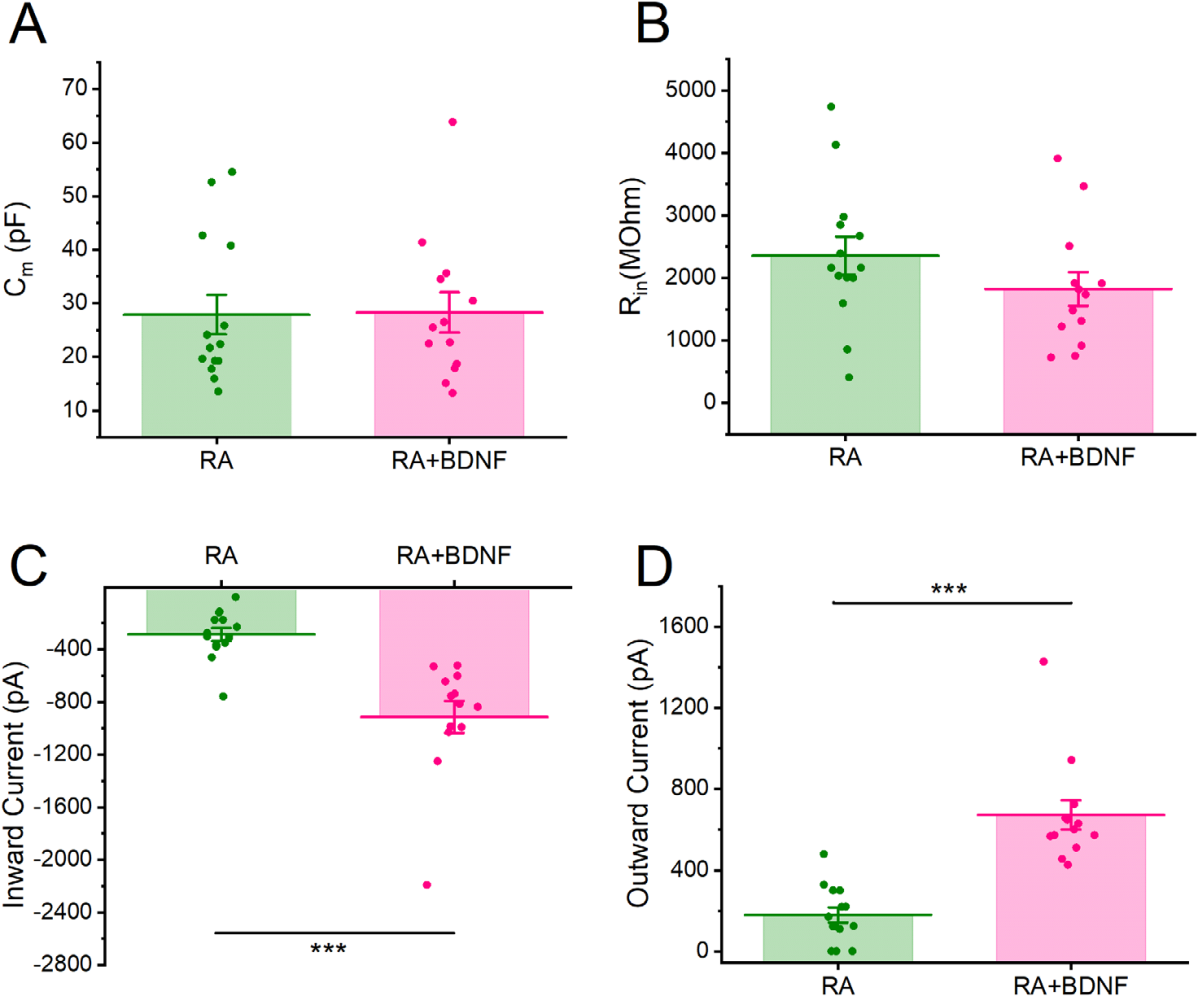
Quantification of the differences in biophysical properties of SH‐SY5Y‐derived neurons treated with two different differentiation protocols. (A–D) Bar charts showing the differences in membrane capacitance (C_m_) (A), input resistance (R_in_) (B), maximal inward current (C), and maximal outward current at +50 mV (D) between cells treated for 20 days with RA (RA) and cells treated for 7 days with RA followed by 4 days with BDNF (RA + BDNF). Data are represented as mean ± s.e.m. and dots are individual cells. The statistical test used to compare the two groups is the two‐sample *t*‐test. *p < 0.05; **p < 0.01; ***p < 0.001.

To assess potential differences in the neuronal differentiation outcomes between the two protocols, we compared input resistance (R_in_) and membrane capacitance (C_m_) using a two‐sample *t*‐test at the end of the differentiation period. R_in_ is a passive property of the cell that is inversely related to the number and gating of ionic channels expressed by the cell, while C_m_ depends on the surface area of the membrane and correlates with the dimension of the cell. We did not find significant differences in either C_m_ or R_in_ between the two protocols (Figure 3A,B) (RA: C_m_ = 27.85 ± 3.69 pF, R_in_ = 2354.39 ± 301.99 MΩ, *N* = 14 vs. RA + BDNF: C_m_ = 28.29 ± 3.77 pF, R_in_ = 1822.24 ± 271.14 MΩ, *N* = 13; relative to C_m_: DF = 25, *t*‐value = −0.0826, *p* = 0.9341; relative to R_in_: DF = 25, *t*‐value = 1.3039, *p* = 0.2041).

We then measured the amplitude of maximal inward and outward currents (Figure 2C,D) as their expression is crucial for the generation of APs (Figure 2E,F). Both inward and outward currents were significantly lower in cells differentiated with RA alone compared to those treated with RA + BDNF (RA: inward current = −286 ± 49.38 pA, outward current = 178.52 ± 37.56, *N* = 14 vs. RA + BDNF: inward current = −914.24 ± 122.04 pA, outward current = 672.64 ± 72.61, *N* = 13; relative to inward current: DF = 25, *t*‐value = 4.8973, *p* = 4.8619 · 10^−5^; relative to outward current: DF = 25, *t*‐value = −6.1741, *p* = 1,8636 · 10^−6^; ***p < 0.001) as shown in Figure 3C,D. Consistently, cells differentiated with RA generally exhibited abortive APs (defined as APs with amplitudes lower than 25 mV) when stimulated with supra‐threshold current steps (Figure 2E). In contrast, cells treated with both RA and BDNF were capable of generating single or repetitive firing patterns as shown in Figure 2F.

Overall, these findings indicate that the RA + BDNF differentiation protocol is more effective in promoting the neuronal differentiation of SH‐SY5Y cells. Therefore, all subsequent experiments were conducted using this differentiation protocol.

### 3.2 Effect of VA945 on Voltage‐Dependent Na^+^ Currents of SH‐SY5Y‐Derived Neurons

With the aim of investigating the effect of VA945 on voltage‐dependent Na^+^ currents, we applied the compound via focal perfusion to SH‐SY5Y differentiated neurons.

**FIGURE 4:**
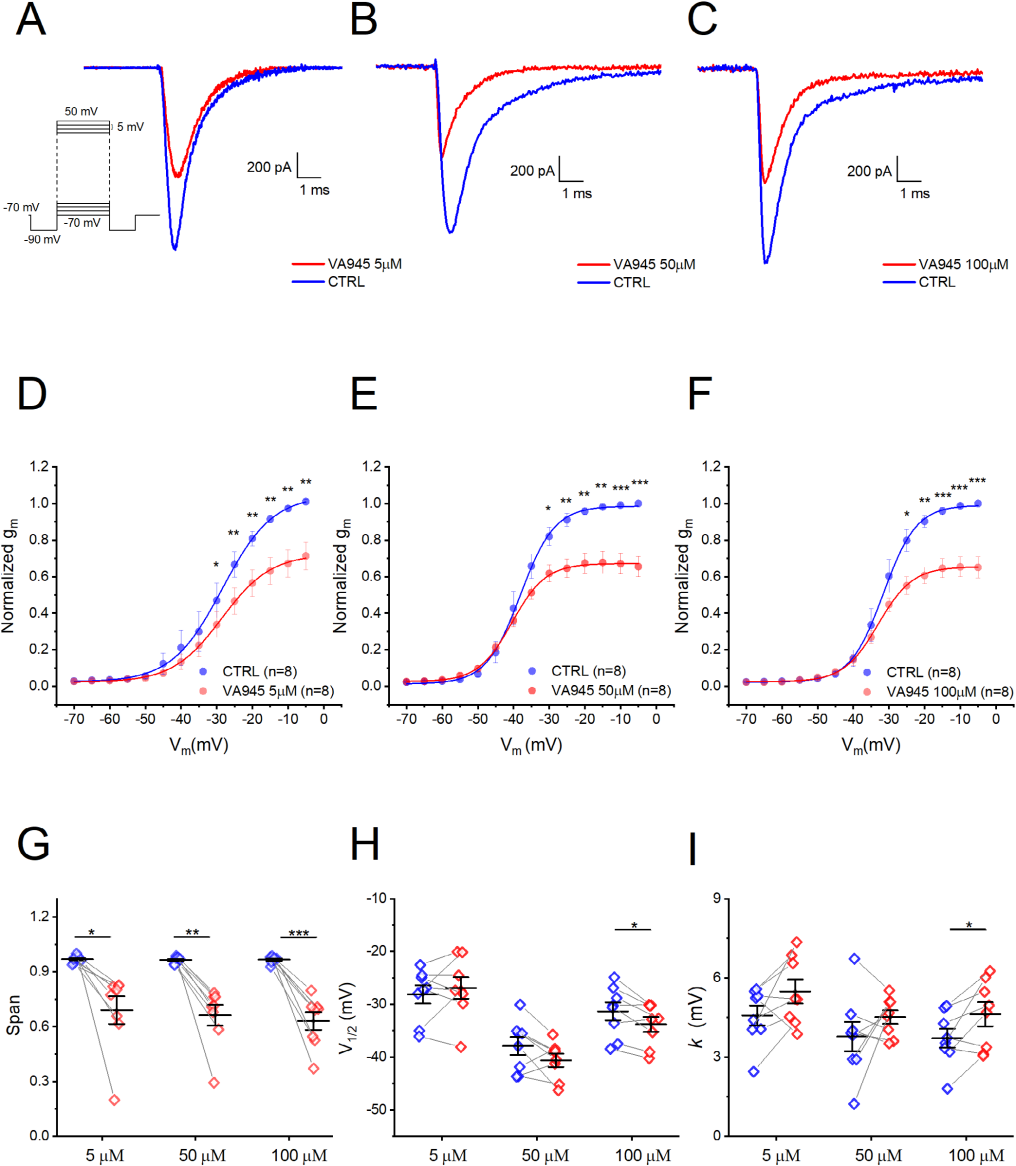
Effect of VA945 on activation of voltage‐dependent Na^+^ currents. (A–C) Representative sample traces of voltage‐dependent Na^+^ currents recorded in voltage‐clamp at −20 mV, showing the reduction of the amplitude with the perfusion of VA945 at different concentrations: 5 μM (A), 50 μM (B), 100 μM (C). The stimulation protocol is shown as an inset in panel A. (D–F) Normalized mean activation curves in CTRL (blue) and in presence of VA945 (red) at three different concentrations: 5 μM (D), 50 μM (E), 100 μM (F). At all concentrations, it is visible a reduction in the conductance g_m_ following the perfusion of the drug. (G–I) Scatter plots comparing in CTRL (blue) and VA945 (red) the parameters of span (G), V_1/2_ (H) and k (I) derived by fitting the normalized activation curves of single cells with the Boltzmann equation described in “materials and methods”. Data are represented as mean ± s.e.m. and dots (in G–I) represent individual cells. Statistical significance was analyzed with paired *t*‐test: *p < 0.05; **p < 0.01; ***p < 0.001.

Voltage‐gated sodium currents were pharmacologically isolated by blocking potassium and calcium voltage‐gated currents (see Table 1 and Material and Methods section, combination of solutions 2). Recordings were performed using the whole‐cell patch‐clamp technique. To evoke Na^+^‐currents, cells were initially hyperpolarized at −90 mV for 20 ms from a holding potential of −70 mV. Subsequently a series of 40 ms depolarizing voltage steps from −70 mV to +50 mV in 5 mV increments were applied. (Figure 4A inset). We tested three different concentrations of VA945: 5 μM (Figure 4A), 50 μM (Figure 4B), 100 μM (Figure 4C). In all cases, focal perfusion of the drug resulted in a clear reduction of the peak current. To quantify the observed reductions, we compared normalized activation curves, as detailed in the Material and Methods section. Briefly, current density was calculated by dividing the amplitude of the peak current recorded at each depolarizing step by the C_m_ of the corresponding cell. Conductance (G_m_) was computed as the ratio between the current density at each membrane potential and the corresponding driving force, with the Na^+^ reversal potential (E_Na_) estimated at +68.48 mV based on our ionic conditions. Finally, the conductance values were normalized to the maximal conductance for each cell and plotted against membrane potential to obtain the normalized activation curves (Figure 4D–F). We found a significant reduction in normalized conductance (g_m_) across different potentials, depending on the concentration of VA945. Specifically, at 5 μM and 50 μM, significant differences were detected between −30 and −5 mV (*N* = 8 for each group; *p < 0.05, **p < 0.01, respectively). At 100 μM, a significant reduction was observed between −25 and −5 mV (*N* = 8; ***p < 0.001) (Figure 4D–F; All statistical parameters are shown in S1). To further analyze the effects of VA945 on Na^+^ channel activation, we then fitted the Na^+^ activation curves using a Boltzmann equation to extrapolate key parameters including (i) span (defined as the difference between the maximum and minimum value of conductance), (ii) V_1/2_, (voltage value at which half maximal activation occurs), (iii) *k*, the slope factor (defined as the voltage corresponding to an e‐fold increase in conductance). For all three concentrations, VA945 caused a significant reduction in the span respectively of 27.83% ± 7.99% (5 μM; DF = 7, *t*‐value = 3.4807, *p* = 0.0103, *p < 0.05), 30.23% ± 5.73% (50 μM; DF = 7, *t*‐value = 5.2751, *p* = 0.0012, **p < 0.01), and 33.54% ± 4.94% (100 μM; DF = 7, *t*‐value = 6.7874, *p* = 2.5612 · 10^−4^, ***p < 0.001) (Figure 4G). We found no significant difference in the V_1/2_ following treatment with 5 μM (control condition (**CTRL**): V_1/2_ = −28.12 ± 1.73 mV, **VA945**: V_1/2_ = −26.91 ± 2.04 mV; DF = 7, *t*‐value = −0.7789, *p* = 0.4616) and 50 μM (**CTRL**: V_1/2_ = −37.87 ± 1.70 mV, **VA945**: V_1/2_ = −40.61 ± 1.25 mV; DF = 7, *t*‐value = 1.9127, *p* = 0.0974) of VA945. However, perfusion with 100 μM VA945 resulted in a significant, shift toward more negative potentials (**CTRL**: V_1/2_ = −31.34 ± 1.70 mV, **VA945**: V_1/2_ = −33.80 ± 1.42 mV; DF = 7, *t*‐value = 3.1644, *p* = 0.0158, *p < 0.05) (Figure 4H). The slope factor (*k*) remained unchanged at both 5 μM (**CTRL**: *k* = 4.58 ± 0.38 mV, **VA945**: *k* = 5.49 ± 0.45 mV; DF = 7, *t*‐value = −1.8661, *p* = 0.1043) and 50 μM (**CTRL**: *k* = 3.78 ± 0.56 mV, **VA945**: *k* = 4.52 ± 0.26 mV; DF = 7, *t*‐value = −1.2545, *p* = 0.2499). In contrast, treatment with 100 μM resulted in a significant increase in *k* (**CTRL**: *k* = 3.71 ± 0.36 mV, **VA945**: *k* = 4.62 ± 0.46 mV; DF = 7, *t*‐value = −2.7389, *p* = 0.0290, *p < 0.05), indicating a reduction in the steepness of the voltage‐dependence curve (Figure 4I).

**FIGURE 5:**
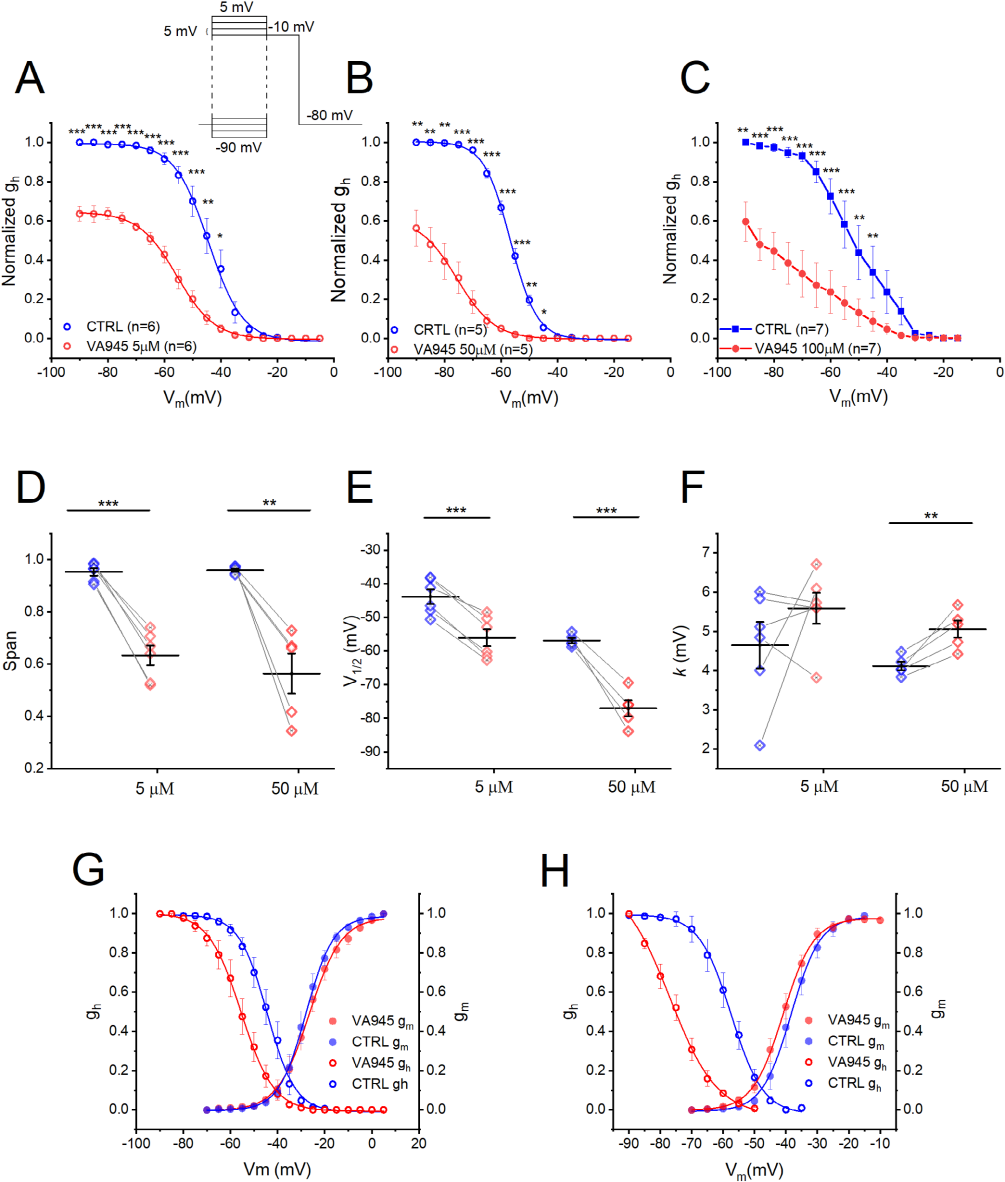
Effect of VA945 on inactivation of voltage‐dependent Na^+^ currents. (A–C) Normalized mean inactivation curves in CTRL (blue) and with VA945 perfusion (red) at 5 μM (A), 50 μM (B) and 100 μM (C). The curves show a significant reduction in the maximal conductance (G_max_) and a shift toward more hyperpolarized potentials at all three tested concentrations. The stimulation protocol is shown as an inset in panel A. (D–F) Scatter plots comparing the parameters of span (D), V_1/2_ (E), *k* (F) derived by fitting the normalized inactivation curves of single cells with the Boltzmann equation (detailed in “materials and methods”) in CTRL (blue) and VA945 (red). (G, H) Window currents were calculated as the area under the activation and inactivation curves in CTRL (blue) and in presence of VA945 (red) at two different concentrations of 5 μM (G) and 50 μM (H). Data are presented as mean ± s.e.m. Dots (in D‐F) represent individual cells. Statistical significance was analyzed with a paired *t*‐test: *p < 0.05; **p < 0.01; ***p < 0.001.

We then investigated the effect of VA945 on steady‐state inactivation of voltage‐dependent Na^+^ currents. The normalized conductance of inactivation was calculated by measuring the Na^+^ peak currents in response to a test depolarizing pulse to −10 mV (24 ms), following a series of conditioning pre‐pulses (40 ms) incremented from −90 to 5 mV in 5 mV steps (Figure 5A inset). Similar to the activation, we observed a significant reduction in the normalized conductance (g_h_) across all three concentrations. Specifically, reductions were seen between −90 and −40 mV at 5 μM: (*N* = 6); between −90 and −45 mV at 50 μM (*N* = 5) and between −90 and −45 mV at; 100 μM (*N* = 7), (*p < 0.05, **p < 0.01, ***p < 0.001). All statistical parameters are shown in S2. In addition, VA945 induced a shift to the left in the inactivation curve at all tested concentrations (Figure 5A–C). Fitting the inactivation data with a Boltzmann equation allowed us to analyze more deeply the functional effect of VA945. Due to the pronounced leftward shift in the inactivation curve at 100 μM, reliable fitting was not feasible at this concentration (Figure 5C); thus, analysis was limited to 5 μM and 50 μM. (Figure 5C). The average reduction in maximal conductance (G_max_) was obtained as the difference between span measured in CTRL and in VA945. The reductions were respectively of 31.93% ± 2.68% (5 μM; DF = 5, *t*‐value = 11.9374, *p* = 7.2719 · 10^−5^, ***p < 0.001) and 39.57% ± 8.02% (50 μM; DF = 4, *t*‐value = 4.9335, *p* = 0.0079, **p < 0.01) (Figure 5D). In addition, a significant leftward shift of the inactivation curve was observed at both concentrations: 12.22 ± 1.11 mV at 5 μM VA945 (**CTRL**: V_1/2_ = −43.80 ± 2.16, **VA945**: V_1/2_ = −56.03 ± 2.53; DF = 5, *t*‐value = 10.9809, *p* = 1.0896 · 10^−4^, ***p < 0.001) and 20.14 ± 2.17 mV at 50 μM (**CTRL**: V_1/2_ = −56.88 ± 0.79, **VA945**: V_1/2_ = −77.03 ± 2.38; DF = 4, *t*‐value = 9.2952, *p* = 7.4530 · 10^−4^, ***p < 0.001) (Figure 5E). Additionally, the slope factor *k* increased significantly at 50 μM of VA945 (**CTRL**: *k* = 4.11 ± 0.11 mV, **VA945**: *k* = 5.06 ± 0.22 mV; DF = 4, *t*‐value = −5.0773, *p* = 0.0071, **p < 0.01), but not of 5 μM (**CTRL**: *k* = 4.65 ± 0.59 mV, **VA945**: *k* = 5.59 ± 0.39 mV; DF = 5, *t*‐value = −1.1683, *p* = 0.2953) (Figure 5F). To assess the reduction in Na^+^ channels availability across different membrane potentials (V_m_), we estimated the window currents by overlaying activation and inactivation curves under CTRL conditions and in the presence of VA945 at 5 μM (Figure 5G) and 50 μM (Figure 5H). The available fraction of channels at each potential, represented by the area of overlap between activation and inactivation curves, was compared between conditions. The reduction in channel was quantified by calculating the difference between the area under the CTRL curve (blue) and the VA945 (red) curve. This analysis revealed a 43% reduction in window current with 5 μM VA945 (Figure 5G) and 63% reduction with 50 μM (Figure 5H).

**FIGURE 6:**
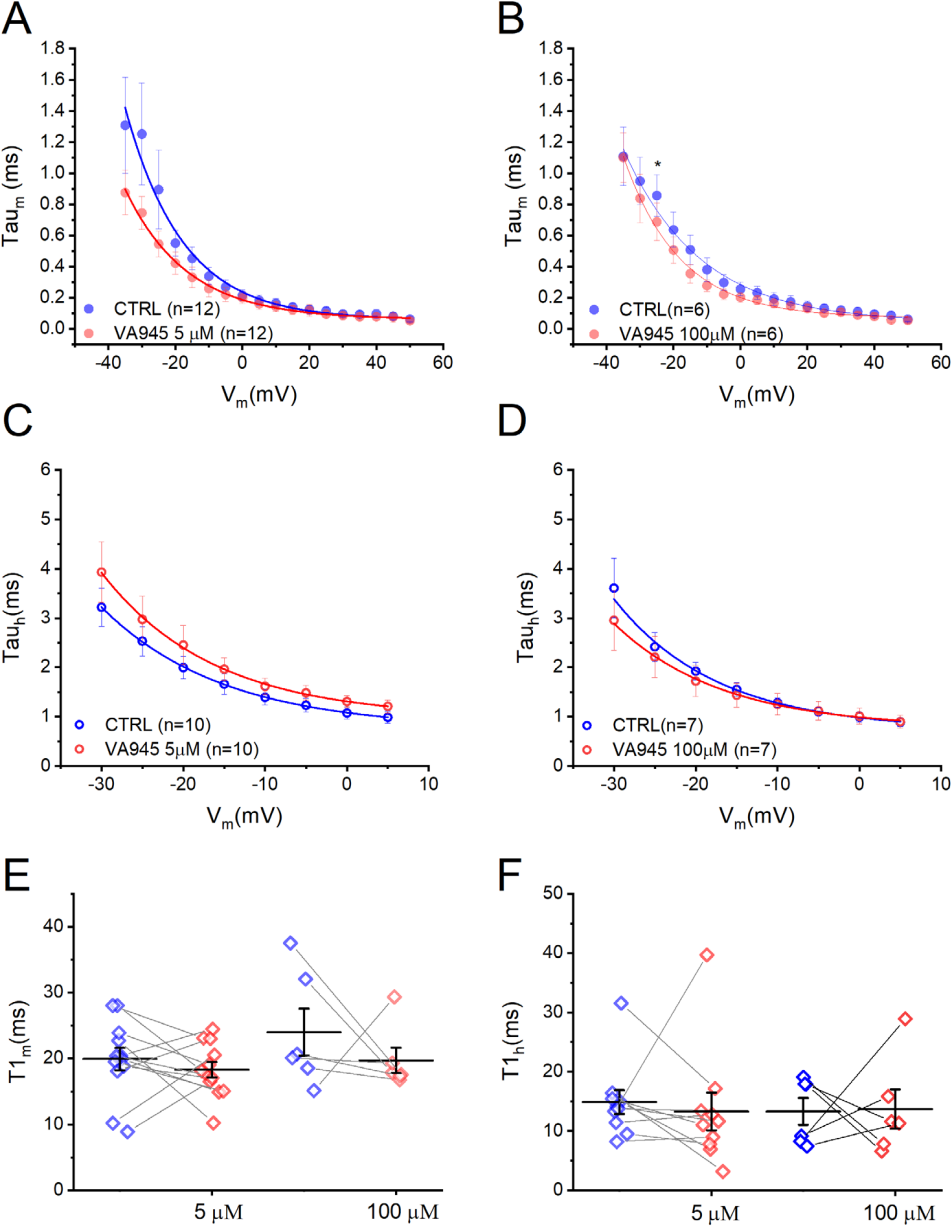
Effect of VA945 on kinetic parameters of voltage‐dependent Na^+^ currents. (A, B) Mean τ_m_‐to voltage relationship in CTRL (blue) and after the perfusion of VA945 (red) at 5 μM (A) and 100 μM (B). (C, D) Mean τ_h_‐to voltage relationship in CTRL (blue) and with the perfusion of VA945 (red) at 5 μM (C) and 100 μM (D). (E, F) Scatter plots comparing CTRL (blue) and VA945 (red) of T1_m_ (E) and T1_h_ (F) obtained by fitting tau‐to‐voltage relationship with an exponential function (as detailed in Materials and Methods). Data are presented as mean ± s.e.m. and dots (in E‐F) represent individual cells. Statistical significance was analyzed with a paired *t*‐test: *p < 0.05; **p < 0.01; ***p < 0.001.

In addition, we investigated the effect of VA945 on the activation and inactivation kinetics properties of Nav channels at the concentration of 5 μM and 100 μM. The activation time constant (τ_m_) was estimated over a voltage range of −40 mV to +50 mV, while the inactivation time constant (τ_h_) was estimated over a voltage range of −30 mV and +5 mV. We found no significant difference in τ_m_ at both concentrations (5 μM: *N* = 10; 100 μM: *N* = 6) (Figure 6A,B). Similarly, τ_h_ remained unchanged at both tested VA945 concentrations (5 μM: *N* = 12; 100 μM: *N* = 7) (Figure 6C,D). The tau‐to‐voltage relationships were fitted with a decay exponential function, as described in the Materials and Methods section. This analysis provided T1_m_ and T1_h_, parameters that reflect the voltage dependency of τ_m_ and τ_h_, respectively. At the concentration of 5 μM, both T1_m_ and T1_h_ were not significantly altered (**CTRL**: T1_m_ = 19.93 ± 1.69 ms and T1_h_ = 14.87 ± 2.03 ms, **VA945**: T1_m_ = 18.31 ± 1.17 ms and T1_h_ = 13.26 ± 3.18 ms; regarding T1_m_: DF = 11, *t*‐value = 0.8064, *p* = 0.4371; regarding T1_h_: DF = 9, *t*‐value = 0.4489, *p* = 0.6641) and at 100 μM (**CTRL**: T1_m_ = 24.02 ± 3.57 ms and T1_h_ = 13.30 ± 2.26 ms, **VA945**: T1_m_ = 19.72 ± 1.96 ms and T1_h_ = 13.69 ± 3.32; regarding T1_m_: DF = 5, *t*‐value = 0.8872, *p* = 0.4156; regarding T1_h_: DF = 5, *t*‐value = −0.0763, *p* = 0.9421).

Overall, these findings suggest that VA945 exerted a significant inhibitory effect on Na_v_ channels in SH‐SY5Y differentiated into neurons using the RA + BDNF protocols. VA945 induces a consistent reduction in the maximal sodium conductance across all tested concentrations (5, 50, and at 100 μM), accompanied by shifts in the activation (at 100 μM) and inactivation (at 5, 50, and at 100 μM) curves to more negative potentials, thereby reducing channel availability at membrane potentials where they are usually recruited and activated. Furthermore, the observed increase in the slope factor (*k*) in the presence of VA945 of the activation (at 100 μM) and inactivation (at 5, 50, and at 100 μM) curves, further indicates a notable decrease in the voltage sensitivity of Na^+^ channels, which may alter the overall excitability of the cells expressing these channels. Altogether, these results underscore the pharmacological potential of VA945 as a modulator of Na^+^ channel activity and, therefore, of the overall neuronal excitability.

### 3.3 Effect of VA945 on Delayed‐Rectifier Voltage‐Dependent K^+^ Currents Expressed by SH‐SY5Y‐Derived Neurons

**FIGURE 7:**
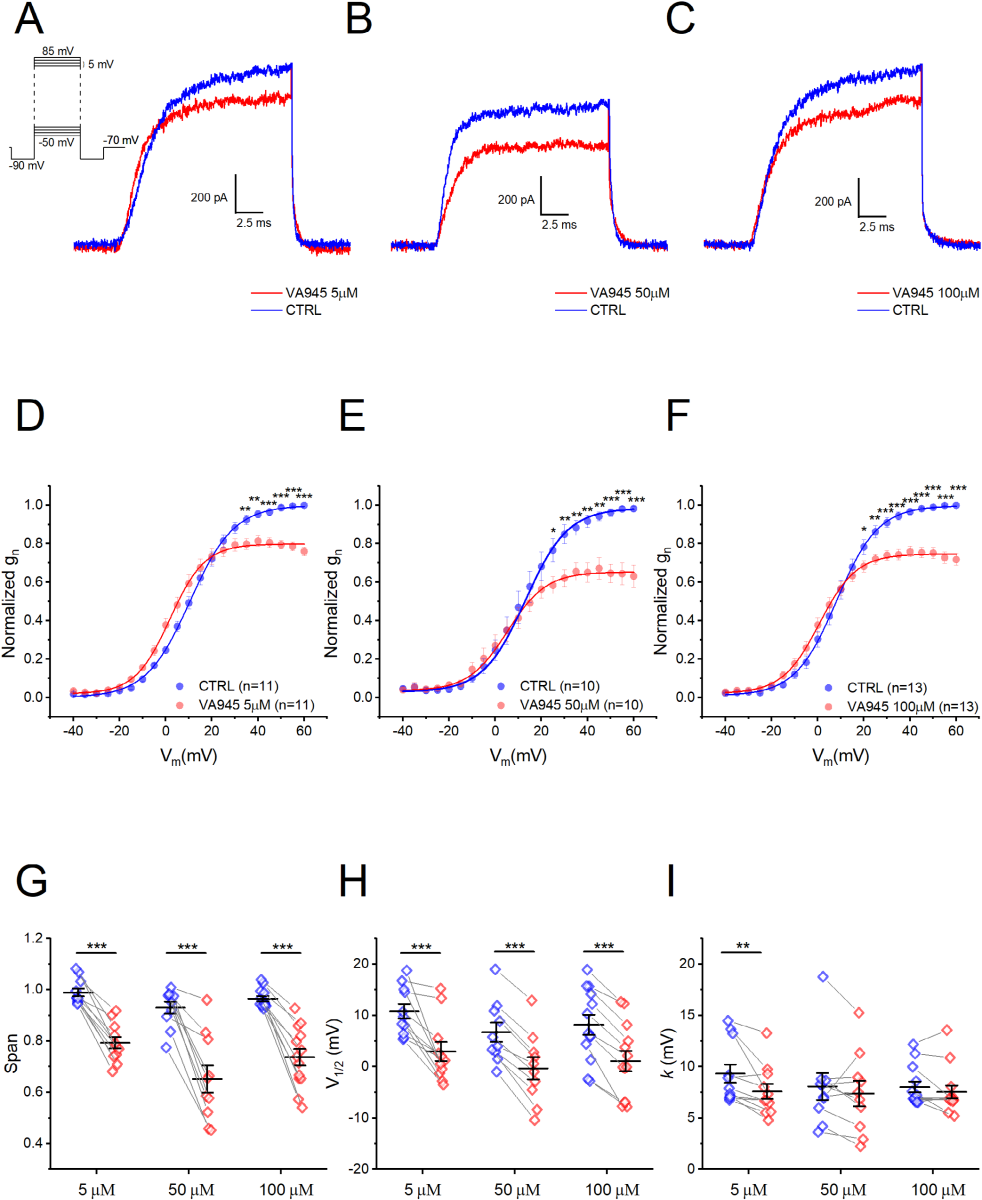
Effect of VA945 on activation of delayed‐rectifier voltage‐dependent K^+^ currents. (A–C) Representative sample traces recorded in voltage‐clamp at 60 mV show a reduction in the delayed‐rectifier K^+^ current amplitude with the perfusion of VA945 at the three different concentrations 5 μM (A), 50 μM (B), 100 μM (C). The stimulation protocol is shown as an inset in panel A. (D–F) Normalized mean activation curves in CTRL (blue) and in the presence of VA945 (red) at three concentrations: 5 μM (D), 50 μM (E), 100 μM (F). At all three concentrations, it is visible a reduction in the maximal conductance (G_max_) following the perfusion of the drug, along with a shift to more hyperpolarized potentials. (G–I) Scatter plots comparing the parameters of span (G), V_1/2_ (H), *k* (I) in CTRL (blue) and VA945 (red), These parameters were derived by fitting the normalized activation curves of single cells with the Boltzmann equation as described in “materials and methods”. Data are presented as mean ± s.e.m. and dots (in G–I) represent individual cells. Statistical significance was analyzed with a paired *t*‐test: *p < 0.05; **p < 0.01; ***p < 0.001.

We recorded isolated delayed‐rectifier voltage‐dependent K^+^ currents while pharmacologically blocking voltage‐gated Na^+^ and Ca^2+^ channels using a combination of 3 solutions as detailed in Table 1 of the Materials and Methods section. Delayed‐rectifier K^+^ currents were elicited by iteratively applying depolarizing steps of 16 ms, from a V_hold_ of −90 to +85 mV, in 5 mV increments (Figure 7A inset). For each step, the current amplitude at steady‐state was normalized by membrane capacitance (C_m_) to obtain current density values. Focal perfusion of VA945 at three different concentrations: 5 μM (Figure 7A), 50 μM (Figure 7B), and 100 μM (Figure 7C) led to a clear reduction in the K^+^ current amplitude. The normalized activation curve was derived from conductance values with the K^+^ reversal potential (E_K_) estimated at −94.55 mV under our experimental conditions. A significant reduction in normalized conductance (g_n_) was observed after perfusion with 5 μM, 50 μM, and 100 μM of VA945 (5 μM: *N* = 11, between 35 and 60 mV; 50 μM: *N* = 10, between 25 and 60 mV; 100 μM: *N* = 13, between 20 and 60 mV; *p < 0.05, **p < 0.01, ***p < 0.001) (Figure 7D–F). By fitting the activation curves with a Boltzmann equation, we extrapolated (i) span, (ii) V_1/2_, and (iii) *k*. At all three concentrations of VA495, we observed a significant reduction in the span: 19.71% ± 2.01% (5 μM; DF = 10, *t*‐value = 9.7850, *p* = 1.9392 · 10^−6^), 27.82% ± 4.22% (50 μM; DF = 9, *t*‐value = 6.5930, *p* = 1.0008 · 10^−4^), and 22.9% ± 2.60% (100 μM; DF = 12, *t*‐value = 8.6888, *p* = 1.5985 · 10^−6^) (Figure 7G). All statistical parameters are shown in S3. Additionally, the activation curves of K^+^ channels were significantly leftward shifted at all concentrations of VA945, as indicated by the changes in the V_1/2_ values: at 5 μM V_1/2_ shift = 7.89 ± 1.69 mV (**CTRL**: V_1/2_ = 10.87 ± 1.42 mV; **VA945**: V_1/2_: 2.98 ± 1.86 mV; DF = 10, *t*‐value = 4.6607, *p* = 8.9334 · 10^−4^), at 50 μM shift = 7.04 ± 0.72 mV (**CTRL**: V_1/2_ = 6.74 ± 1.88 mV, **VA945**: V_1/2_: −0.3 ± 2.15 mV; DF = 9, *t*‐value = 9.8093, *p* = 4.1991 · 10^−6^), and at 100 μM shift = 7.05 ± 0.85 mV (**CTRL**: V_1/2_ = 8.17 ± 1.95 mV, **VA945**: V_1/2_ = 1.12 ± 1.20 mV; DF = 12, *t*‐value = 8.3001, *p* = 2.5738 · 10^−6^) (***p < 0.001) (Figure 7H). Regarding the slope factor *k* we found no significant difference at 50 μM (**CTRL**: *k =* 8.08 ± 1.32 mV, **VA945**: *k =* 7.40 ± 1.25 mV; DF = 9, *t*‐value = 0.9676, *p* = 0.3585) and 100 μM (**CTRL**: *k =* 8.02 ± 0.53 mV, **VA945**: *k* = 7.55 ± 0.63 mV; DF = 12, *t*‐value = 1.336, *p* = 0.206). However, at 5 μM, **VA495** significantly decreased *k* (**CTRL**: *k =* 9.34 ± 0.9 mV, **VA945**: *k* = 7.61 ± 0.73 mV; DF = 10, *t*‐value = 3.2040, *p* = 0.0094; **p < 0.01) indicating an increased steepness in voltage‐dependence (Figure 7I).

**FIGURE 8:**
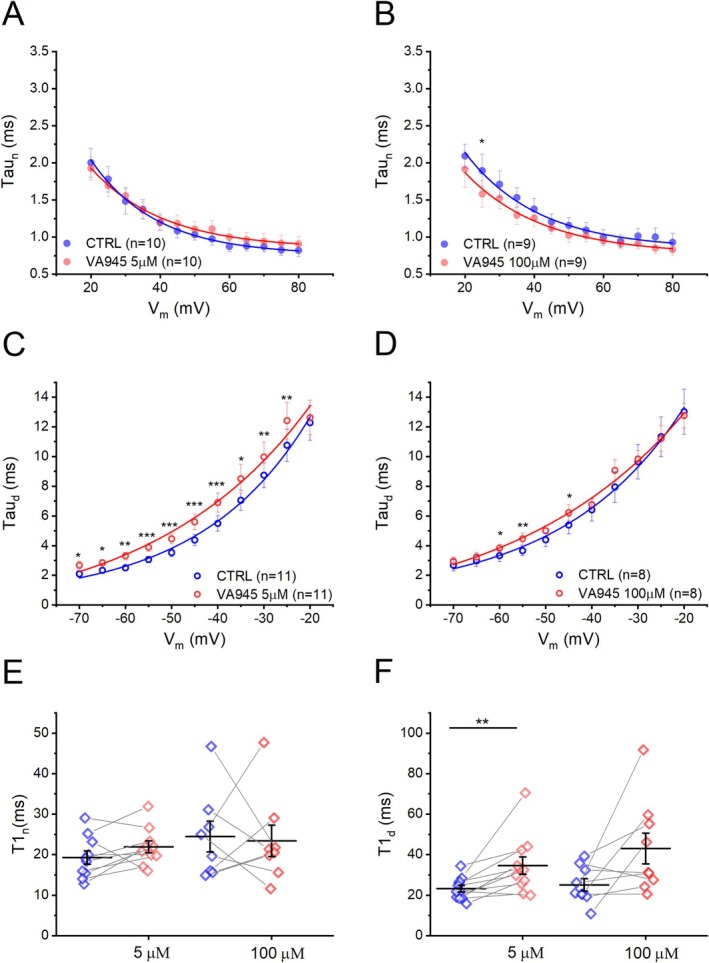
Effect of VA945 on kinetic parameters of delayed‐rectifier voltage‐dependent K^+^ currents. (A, B) Mean τ_n_‐to voltage relationship in CTRL (blue) and with the perfusion of VA945 (red) at 5 μM (A) and 100 μM (B). (C, D) Mean τ_d_‐to voltage relationship in CTRL (blue) and with the perfusion of VA945 (red) at 5 μM (C) and 100 μM (D). (E, F) Scatter plots comparing T1_n_ (E) and T1_d_ (F) in CTRL (blue) and VA945 (red) obtained by fitting tau‐to‐voltage relationship with an exponential function. Data are represented as mean ± s.e.m. and dots (in E‐F) represent individual cells. Statistical significance was analyzed with a paired *t*‐test: *p < 0.05; **p < 0.01; ***p < 0.001.

Subsequently, we analyzed the kinetic parameters of activation (τ_n_) and deactivation (τ_d_) of the delayed‐rectifier K^+^ channels following treatment with 5 μM and 100 μM VA945. No significant difference was observed in τ_n_ at both tested concentrations (5 μM: *N* = 10; 100 μM: *N* = 9) (Figure 8A,B). In contrast, τ_d_ was significantly slower at 5 μM across all examined potentials (Figure 8C). At 100 μM, a significant difference in τ_d_ was observed only at −60, −55, and −45 mV (Figure 8D) (5 μM: *N* = 11; 100 μM: *N* = 8. *p < 0.05, **p < 0.01, ***p < 0.001). All statistical parameters are shown in S4. To further characterize the voltage‐dependence of τ_n_ and τ_d_, we derived the parameters T1_n_ and T1_d_, by fitting the tau‐to‐voltage relationship with exponential functions, as described in Materials and Methods. T1_n_ was not significantly affected at either concentration (5 μM **CTRL**: T1_n_ = 19.31 ± 1.64, **VA945**: T1_n_ = 21.92 ± 1.46, DF = 9, *t*‐value = −1.7241, *p* = 0.1187; 100 μM **CTRL**: T1_n_ = 24.47 ± 3.80, **VA945**: T1_n_ = 23.42 ± 3.89, DF = 7, *t*‐value = 0.1980, *p* = 0.8486) (Figure 8E). However, T1_d_ was significantly increased at 5 μM (**CTRL**: T1_d_ = 23.23 ± 1.64, **VA945**: T1_d_ = 34.60 ± 4.27; DF = 10, *t*‐value = −3.1702, *p* = 0.00998, **p < 0.01), but not at 100 μM (**CTRL**: T1_d_ = 25.08 ± 3.04, **VA945**: T1_d_ = 43.01 ± 7.63; DF = 8, *t*‐value = −2.0974, *p* = 0.0692) (Figure 8F), suggesting a modulation of K+ channel deactivation kinetics more pronounced at low concentrations of the drug.

Overall, these results demonstrate that delayed‐rectifier voltage‐dependent K^+^ channels are modulated by VA945, as indicated by the decrease in their maximal conductance (G_max_) at all three tested concentrations. Moreover, the shift in V_1/2_ suggests that these K^+^ channels are recruited at more hyperpolarized potentials when exposed to VA945. Additionally, at lower concentrations VA945 decreases the slope factor *k*, enhancing the voltage sensitivity of these channels. The observed slowdown of τ_d_, thereby suggests that VA945 affects also the deactivation kinetics of these channels. Collectively, these results highlight the capability of VA945 to greatly influence neuronal excitability by modulating voltage‐gated K^+^ channels which are critical elements of neuronal firing and are involved in the cellular and molecular signaling pathways that govern neuronal survival and apoptosis.

## 4 Discussion

NDDs are characterized by a progressive loss of neurons and synaptic connections, which leads to functional decline and deficits in cognition, motor skills, and sensory processing. A major factor contributing to neurodegeneration is excitotoxicity, a pathological process caused by the excessive activation of excitatory receptors. This overstimulation is typically driven by an increased or prolonged release of excitatory neurotransmitters, primarily glutamate (Armada‐Moreira et al. 2020).

The amplified or prolonged activation of glutamate receptors triggers a cascade of events, including cationic influx, mitochondrial dysfunction, energetic and oxidative stress, and overproduction of reactive oxygen species (ROS), ultimately leading to neuronal death (Connolly and Prehn 2015; Lipton et al. 1993; Piña‐Crespo et al. 2014; Prentice et al. 2015). Several neuroprotective and anticonvulsant drugs, including riluzole (Rilutek), target excitotoxicity‐related pathways, to provide an effective treatment against neurodegeneration (Bellingham 2011; Binvignat and Olloquequi 2020; Bryson et al. 1996).

Riluzole exerts its neuroprotective effects through multiple mechanisms, including the modulation of both inward and outward voltage‐dependent currents involved in the development of excitotoxicity (Bellingham 2011; Beltran‐Parrazal and Charles 2003; Doble 1996; Smith et al. 2024; Zona et al. 1998). Specifically, it affects both voltage‐dependent Na^+^ currents and delayed‐rectifier K^+^ currents, both of which are crucial for AP generation and propagation, as well as for the control of neuronal excitability (Bellingham 2011; Errington et al. 2005; Prakriya and Mennerick 2000; Taylor and Meldrum 1995; Zona et al. 1998). By reducing the amplitude of the voltage‐dependent Na^+^ currents, riluzole can impair the depolarization required for the propagation of the APs along the axon (Abdelsayed and Sokolov 2013; Farber et al. 2002). As a result, the inhibition of AP propagation is expected to prevent the synaptic release of neurotransmitter, thereby reducing excitotoxic neuronal damage.

Riluzole decreases the peak Na^+^ current and shifts the voltage‐dependence of inactivation of rapidly inactivating Na^+^ channels toward more hyperpolarized potentials. Moreover, it has been shown that riluzole inhibits the persistent Na^+^ current (I_NaP_) in mammalian central nervous system (CNS) neurons (Beltran‐Parrazal and Charles 2003; Theiss et al. 2007; Urbani and Belluzzi 2000; Wu et al. 2005). Recent studies have also demonstrated that riluzole helps to stabilize a specific state of voltage‐gated Na^+^ channels known as the incomplete inactivation state. This state underlies the late Na^+^ current (I_NaL_), which contributes to the hyperexcitability observed in motor neurons affected by NDDs such as ALS. As a result, by targeting this mechanism riluzole reduces the pathological overexcitability of these neurons (Hollingworth et al. 2024).

Additionally, riluzole has a concentration‐dependent effect on the outward K^+^ currents in cultured rat cortical neurons (Zona et al. 1998). While the amplitude of the peak of the transient A‐type K^+^ current I_A_ remains unaffected, the amplitude of the delayed‐rectifier outward K^+^ current significantly decreased during the perfusion of riluzole. This reduction in outward K^+^ currents may prolong the depolarization phase of the AP, thereby promoting the inactivation of Na^+^ channels and increasing the proportion of channels that remain in the inactivated state. This effect leads to a further reduction in neuronal excitability. Riluzole modulates these ion channels in a concentration‐dependent manner: concentrations < 10 μM lead to a decrease in the amplitude of the Na^+^ current, while higher concentrations > 50 μM are required to affect K^+^ current (Bellingham 2011). However, the limitations in the dose‐dependent efficacy of riluzole underscore the need for novel agents that more effectively target excitotoxic pathways while preserving neuronal excitability. Such compounds could serve as improved potential therapeutic tools for treating excitotoxicity in NDDs, including ALS. Based on all these considerations, we investigated the physiological effects of VA945, a new riluzole‐derived compound on voltage‐dependent Na^+^ and K^+^ current in neurons. This study aims to evaluate the potential of VA945 as a multi‐target therapy with enhanced efficacy across a broader range of concentrations.

Our experiments examined in detail the effects of VA945 on voltage‐dependent Na^+^ and delayed‐rectifier K^+^ channels, two key conductances in neuronal excitability. For this study, we employed cultured human neuroblastoma SH‐SY5Y cells differentiated into neurons. The SH‐SY5Y cell line is an immortalized human neuroblastoma line widely used in neuroscience research as a model for neuronal function. Upon differentiation, SH‐SY5Y cells: (i) adopt a neuron‐like morphology, exhibiting a pronounced axonal outgrowth (Bell et al. 2021; Encinas et al. 2000; Kovalevich and Langford 2013; Shipley et al. 2016), (ii) form functional synapses (Cheung et al. 2009; Sarkanen et al. 2007), and (iii) become electrically excitable due to the expression of voltage‐gated Na^+^ and K^+^ channels (Hill et al. 2014; Johansson 1994; Park et al. 2010; Sun et al. 2020; Tosetti et al. 1998). These neuron‐like properties make SH‐SY5Y cells an ideal model for analyzing isolated voltage‐dependent currents (Gambino et al. 2022; Santillo 2022; Toselli et al. 1991; Tosetti et al. 1998). In this study, we compared the efficacy of two different protocols aimed at differentiating SH‐SY5Y glioblastoma cells into neuron‐like cells with the goal of finding the most robust, efficient, and reliable method. Our findings revealed that cells treated with a combination of RA + BDNF exhibited significantly higher amplitudes of both inward and outward currents. This increase suggests an enhanced expression of voltage‐dependent ion channels, indicating a more mature neuronal phenotype of the treated cells. Thus, this RA + BDNF differentiation protocol provides an effective platform for investigating the electrophysiological effects of VA945.

Cells treated with VA945 exhibited a significant decrease in maximum conductance (G_max_) and, consequently, in the amplitude of both transient Na^+^ and delayed‐rectifier K^+^ currents. This suggests that VA945 targets the same key currents as riluzole (Bellingham 2011), potentially providing a similar neuroprotective effect. Notably, this reduction was observed across all three tested concentrations, indicating that VA945 has a broad range of overlapping effects on both target currents. This contrasts with riluzole's concentration‐dependent effects (Bellingham 2011).

We also observed that the effects of VA945 extend to additional properties of these currents, impacting their overall functionality and further emphasizing the multitarget potential of this compound. At a concentration of 100 μM, VA945 induced a rightward shift in the half‐activation voltage V_1/2_ of the voltage‐gated Na^+^ current, accompanied by a shift toward a more hyperpolarized value of the membrane potential in the Na^+^ inactivation curve across all tested concentrations. These shifts indicate that Na^+^ channels activate at more depolarized potentials and inactivate at more hyperpolarized ones, thereby reducing the availability of Na^+^ channels at different membrane potentials. The observed variability in V_1/2_ values for I_Na_ activation and inactivation among control cells is likely attributable to differences in the differentiation state across batches, despite using the same differentiation protocol. However, it is important to point out that the effect of VA945 was assessed using a paired experimental design. Such an experimental approach, where control and VA945‐treated cells were compared within the same batch, effectively minimizes the impact of batch‐to‐batch variability on our conclusions concerning VA945's effects on the biophysical properties of I_Na_ and I_K_. Window current analysis confirmed a narrowed window for Na^+^ channels' activation range, which could likely limit the cell's ability to respond effectively to depolarizing steps, resulting in a reduced AP firing frequency. These findings align with those observed with other neuroprotective and anticonvulsant agents (Bellingham 2013; Wang et al. 2015; Zona et al. 1998). Similarly, we found that the V_1/2_ of the activation curve of the delayed‐rectifier K^+^ channels shifted toward more negative potentials in the presence of VA945 compared to control conditions. This leftward shift could enhance the K^+^ current at subthreshold voltages, thereby increasing the ability of the cell to oppose depolarization and suppress neuronal excitability. By reducing the likelihood of reaching the AP threshold this effect could contribute to reduced firing frequency, an effect shared with other neuroprotective drugs (Zhang et al. 2019).

Additionally, the observed increase in the slope factors of both activation and inactivation curves of the voltage‐gated Na^+^ current at medium to high concentrations of VA945 suggests a reduced voltage sensitivity of these Na^+^ channels. This diminished voltage‐dependence may further attenuate Na^+^‐driven depolarization. While many neuroprotective drugs do not significantly affect this parameter, a slight increase in the slope factor has been reported in cells treated with riluzole and its derivatives (Anzini et al. 2010). In contrast, at low concentrations of VA945, a decrease in the slope factor of the activation curve of the K^+^ delayed‐rectifier current was observed. This change indicates enhanced voltage‐dependence, which may amplify the hyperpolarizing effect of the K^+^ current strengthening its ability to counteract membrane depolarization. Taken together, these complementary shifts might contribute to the anticonvulsant and neuroprotective profile of VA945.

Regarding channel kinetics, VA945 had no effect on the activation kinetics of either Na^+^ or K^+^ channels, and on the Na^+^ inactivation kinetics in line with riluzole's action profile (Bellingham 2011). In contrast, the kinetics of K^+^ deactivation were notably slower with VA945. Such effects were also reported for other anticonvulsant drugs, like retigabine, where the shift of the activation curve to more hyperpolarized potentials and the slowdown of the deactivation kinetics contribute to open K^+^ channels and reduce firing frequency (Main et al. 2000). These findings suggest that VA945 may exert similar actions, contributing to its observed neuroprotective and anticonvulsant effects.

Given VA945's multi‐target effects on excitability‐related ion currents, this compound shows significant promise for further in vitro and in vivo investigations, particularly in ALS models. Additionally, VA945 could also have potential as an anticonvulsant agent, warranting exploration in epilepsy and stroke models. The therapeutic relevance of this compound is further supported by its demonstrated ability to cross the blood–brain barrier (BBB) and to mitigate stress‐induced conditions (Maramai et al. 2024). Therefore, it will be essential and highly relevant to perform additional studies on both acute and chronic administration of this compound across different cellular and animal models of neurodegenerative diseases, including ALS. Such investigations will provide valuable insights into the therapeutic efficacy of VA945 and support the translational potential for clinical applications.

## Author Contributions

**J. Cazzola:** investigation, data curation, formal analysis, methodology, writing – review and editing. **F. Talpo:** investigation, data curation, formal analysis, writing – review and editing. **G. Faravelli:** formal analysis, writing – original draft. **C. Donati:** writing – original draft, formal analysis. **S. Maramai:** conceptualization, visualization, writing – review and editing, resources. **M. Saletti:** resources, conceptualization, visualization, writing – review and editing. **G. Giuliani:** conceptualization, visualization, writing – review and editing, resources. **M. Paolino:** visualization, writing – review and editing, conceptualization. **A. Cappelli:** conceptualization, visualization, writing – review and editing, resources. **M. Anzini:** conceptualization, visualization, writing – review and editing, resources. **P. Sommi:** resources, methodology. **A. Vitali:** methodology, resources. **A. Sala:** formal analysis, writing – original draft. **A. Trucco:** writing – original draft, formal analysis. **G. R. Biella:** supervision, conceptualization, funding acquisition, writing – review and editing, project administration. **P. Spaiardi:** conceptualization, funding acquisition, writing – review and editing, supervision, investigation.

## Conflicts of Interest

The authors declare no conflicts of interest.

## Peer Review

The peer review history for this article is available at https://www.webofscience.com/api/gateway/wos/peer‐review/10.1111/jnc.70280.

## Supporting information


**Data S1:** jnc70280‐sup‐0001‐Supinfo1.pdf.

## Data Availability

The data that support the findings of this study are available from the corresponding author upon reasonable request.
