# Supplementary material for: Evaluation of a New Riluzole‐Based Compound VA945 on Sodium and Potassium Conductances Expressed by SH‐SY5Y‐ Derived Neurons
Source: J Neurochem. 2025 Oct 31;169(11):e70280. doi: 10.1111/jnc.70280 (PMC12577522; doi:10.1111/jnc.70280)
Supplement: Supplementary file 1 — Data S1: jnc70280‐sup‐0001‐Supinfo1.pdf. [file JNC-169-0-s001.pdf]

Evaluation of a new riluzole-based compound VA945 on sodium and potassium conductances expressed by SH-SY5Y- derived neurons

J. Cazzola*[1], F. Talpo*[1], G. Faravelli[1], C. Donati[1], S. Maramai[2], M. Saletti[2], G. Giuliani[2], M. Paolino[2], A. Cappelli[2], M. Anzini[2], P. Sommi[3], A. Vitali[4,5], A. Sala[6], A. Trucco[1], G. R. Biella[1,7], P. Spaiardi[1,7]

[1]Department of Biology and Biotechnology "Lazzaro Spallanzani", University of Pavia, Pavia, Italy.

[2]Department of Biotechnology, Chemistry and Pharmacy, University of Siena, Siena I-53100, Italy.

[3]Department of Molecular Medicine, Human Physiology Unit, University of Pavia, 27100 Pavia, Italy.

[4]Department of Chemistry, University of Pavia, 27100 Pavia, Italy.

[5]SC Coordination, support and monitoring of clinical and experimental research- IRCCS Foundation Policlinico San Matteo, 27100 Pavia, Italy.

[6] Department of Molecular Medicine, Biochemistry Unit, University of Pavia, Pavia, Italy.

[7] Istituto Nazionale di Fisica Nucleare, Sezione di Pavia, Pavia, Italy.

*equal contribution

Corresponding author: paolo.spaiardi@unipv.it. https://orcid.org/0000-0001-7418-180X

S.1 Statistical Parameters of Normalized Activation Curves of Sodium Channels. A. DF, *t*-values, and *p*-values at various voltages corresponding to the normalized activation curve shown in Fig. 4D. B. DF, *t*-values, and *p*-values at various voltages corresponding to the normalized activation curve shown in Fig. 4E. C. DF, *t*-values, and *p*-values at various voltages corresponding to the normalized activation curve shown in Fig. 4F. All values are the results of paired *t*-tests.

A. 5 µM

**Voltage (mV)**	**DF**	**t-value**	**p-value**
-30	7	2.482	0.0421
-25	7	3.613	0.0086
-20	7	4.121	0.0044
-15	7	4.371	0.0033
-10	7	4.036	0.0050
-5	7	3.964	0.0054

B. 50 µM

**Voltage (mV)**	**DF**	**t-value**	**p-value**
-30	7	2.912	0.0226
-25	7	4.178	0.0041
-20	7	4.875	0.0018
-15	7	4.845	0.0019
-10	7	5.406	0.0010
-5	7	6.191	0.0004

C. 100 µM

**Voltage (mV)**	**DF**	**t-value**	**p-value**
-25	7	3.150	0.0161
-20	7	5.245	0.0010
-15	7	5.722	0.0007
-10	7	6.195	0.0004
-5	7	6.050	0.0005

S.2 Statistics parameters of normalized inactivation curves of sodium channels. A. DF, *t*-values, and *p*-values at various voltages corresponding to the normalized inactivation curve shown in Fig. 5A. B. DF, *t*-values, and *p*-values at various voltages corresponding to the normalized inactivation curve shown in Fig. 5B. C. DF, *t*-values, and *p*-values at various voltages corresponding to the normalized inactivation curve shown in Fig. 5C. All values are the results of paired *t*-tests.

A. 5 µM

Voltage (mV)	DF	t-value	p-value
-90	5	9.649	0.0002
-85	5	8.598	0.0003
-80	5	9.694	0.0002
-75	5	12.849	0.00005
-70	5	20.090	0.000006
-65	5	18.3	0.000009
-60	5	16.380	0.00001
-55	5	12.968	0.00005
-50	5	10.132	0.0002
-45	5	6.136	0.0017
-40	5	3.710	0.0138

B. 50 µM

Voltage (mV)	DF	t-value	p-value
-90	4	4.747	0.0090
-85	4	6.012	0.0038
-80	4	6.750	0.0025
-75	4	9.030	0.0008
-70	4	15.737	0.00009
-65	4	36.742	0.000003
-60	4	22.273	0.00002
-55	4	11.086	0.0004
-50	4	8.133	0.0012
-45	4	4.201	0.010

C. 100 µM

Voltage (mV)	DF	t-value	p-value
-90	6	4.023	0.0069
-85	6	6.611	0.0006
-80	6	5.910	0.0010
-75	6	6.069	0.0009
-70	6	6.222	0.0008
-65	6	8.433	0.0001
-60	6	8.787	0.0001
-55	6	6.254	0.0008
-50	6	3.734	0.010
-45	6	2.567	0.0425

S.3 Statistics parameters of normalized activation curves of potassium channels. A. DF, *t*-values, and *p*-values at various voltages corresponding to the normalized activation curve shown in Fig. 7D. B. DF, *t*-values, and *p*-values at various voltages corresponding to the normalized activation curve shown in Fig. 7E. C. DF, *t*-values, and *p*-values at various voltages corresponding to the normalized activation curve shown in Fig. 7F. All values are the results of paired *t*-tests.

A. 5 µM

Voltage (mV)	DF	t-value	p-value
35	10	-3.564	0.0052
40	10	4.013	0.0025
45	10	-4.787	0.0007
50	10	6.800	0.00005
55	10	-9.276	0.000003
60	10	10.353	0.000001

B. 50 µM

Voltage (mV)	DF	t-value	p-value
25	9	-2.623	0.023
30	9	-3.493	0.0070
35	9	-3.4	0.0080
40	9	-3.841	0.0040
45	9	-3.819	0.0041
50	9	-5.103	0.0006
55	9	-5.274	0.0005
60	9	-5.203	0.0006

C. 100 µM

Voltage (mV)	DF	t-value	p-value
20	12	2.412	0.033
25	12	3.502	0.0044
30	12	-4.458	0.0008
35	12	5.538	0.0002
40	12	-5.816	0.00008
45	12	7.541	0.000007
50	12	-7.981	0.000004
55	12	9.144	0.0000009
60	12	-9.531	0.0000006

S.4 Statistics parameters of τ_n and τ_d. A. DF, *t*-values, and *p*-values at various voltages corresponding to the τ_n-to voltage relationship shown in Fig. 8B. B. DF, *t*-values, and *p*-values at various voltages corresponding to the τ_d-to voltage relationship shown in Fig. 8C. C. DF, *t*-values, and *p*-values at various voltages corresponding to the τ_d-to voltage relationship curve shown in Fig. 8D. All values are the results of paired *t*-tests.

A. τ_n 100 µM

Voltage (mV)	DF	t-value	p-value
25	7	2.714	0.026

B. τ_d 5 µM

Voltage (mV)	DF	t-value	p-value
-25	10	4.258	0.0017
-30	10	3.388	0.0069
-35	10	-2.896	0.016
-40	10	7.781	0.00002
-45	10	-4.830	0.0007
-50	10	-5.228	0.0004
-55	10	-6.281	0.00009
-60	10	-4.486	0.0012
-65	10	-3.054	0.012
-60	10	-3.138	0.011

C. τ_d 100 µM

Voltage (mV)	DF	t-value	p-value
-45	7	-2.418	0.0462
-55	7	-4.656	0.0023
-60	7	-3.087	0.0176
